# Prevalence of psychiatric disorders among refugees and migrants in immigration detention: systematic review with meta-analysis

**DOI:** 10.1192/bjo.2021.1026

**Published:** 2021-11-15

**Authors:** Irina Verhülsdonk, Mona Shahab, Marc Molendijk

**Affiliations:** Faculty of Social and Behavioural Sciences, Clinical Psychology Department, Leiden University, the Netherlands; Faculty of Social and Behavioural Sciences, Clinical Psychology Department, Leiden University, the Netherlands and Department of Clinical Epidemiology, Leiden University Medical Center, the Netherlands; Faculty of Social and Behavioural Sciences, Clinical Psychology Department, Leiden University, the Netherlands and Leiden University Medical Center, Leiden Institute of Brain and Cognition, the Netherlands

**Keywords:** Refugees, immigration detention, depression, anxiety, PTSD

## Abstract

**Background:**

The number of forced migrants is increasing worldwide. Some governments detain refugees and migrants in immigration detention centres, which is associated with adverse mental health outcomes.

**Aims:**

To estimate prevalence rates of depression, anxiety and post-traumatic stress disorder (PTSD) in child and adult refugees and migrants in immigration detention.

**Method:**

Pre-registered systematic review with meta-analysis (Prospero ID: CRD42020196078).

**Results:**

Systematic searches in Medline, Embase and Web of Science (final search date 1 October 2020) yielded nine eligible studies on the mental health of detained refugees and migrants (total *n =* 630 refugees and migrants, 522 of them in detention, among which 26 were children). For adults, prevalence rates for depression were 68% (95% CI 0.53–0.83%), for anxiety 54% (95% CI 0.36–0.72%) and for PTSD 42% (95% CI 0.22–0.63%). Theoretical comparisons with data from other meta-analyses revealed that prevalence rates and symptom severity were higher in detained, relative to non-detained samples.

**Conclusions:**

Our data show a huge burden of mental health problems in detained refugees and migrants of all ages, also relative to non-detained samples. This suggests that immigration detention independently and adversely affects the mental health of refugees and migrants. This insight should encourage countries to minimise the use of immigration detention and implement alternative measures instead.

## Background

In the past two decades, forced migration and the displacement of people have reached a new high.^1^ Forced by war, civil conflicts, (natural) disasters, persecution or other violations of human rights, 82.4 million people worldwide have been forced to flee their homes by June 2021.^2^ Among them are internally displaced people, refugees and asylum seekers. Internally displaced people, representing the majority of forced migrants (around 48 million people) are those who have been ‘forced or obliged to flee from their home or place of habitual residence, in particular as a result of or in order to avoid the effects of armed conflicts, situations of generalised violence, violations of human rights or natural or human-made disasters’ and have not crossed ‘an internationally recognised State border’.^3^ Refugees are the second largest group among those forcefully displaced (around 20.7 million people).^2^ According to the 1951 Refugee Convention, a refugee is ‘unable or unwilling to return to their country of origin owing to a well-founded fear of being persecuted for reasons of race, religion, nationality, membership of a particular social group, or political opinion’.^4^ The third and smallest group (around 4.1 million people) are asylum seekers.^2^ Whereas refugees are already under a form of protection, asylum seekers are still awaiting a decision whether or not they will be granted protection.^5^ Terms to refer to these groups are used inconsistently and interchangeably. The International Organization for Migration defines irregular migrants as individuals moving ‘outside the regulatory norms of the sending, transit and receiving countries’.^6^ In this article, we will follow the recommended practice of the United Nations High Commissioner for Refugees (UNHCR) and use ‘refugees and migrants’ as a generic term to refer to internally displaced people, refugees, asylum seekers and irregular migrants as defined above.^7,8^ Forced migration in general is associated with adverse mental health outcomes because of different pre-, peri- and post-migration factors.^9,10^

Refugees and migrants may be exposed to potentially traumatic events and other stressful situations that can occur before migration, during migration and after arrival in the receiving country. As a result, refugees and migrants are particularly vulnerable to psychiatric problems, such as symptoms of depression, post-traumatic stress disorder (PTSD) or anxiety.^11–13^ Upon arrival many countries regularly detain refugees and migrants.^14,15^ The International Organization for Migration defines immigration detention as ‘the deprivation of liberty for migration-related reasons’.^16^ According to the UNHCR, confinement that is enforced ‘within a narrowly bounded or restricted location … and where the only opportunity to leave this limited area is to leave the territory’ qualifies as immigration detention, for example in prisons, detention facilities or closed camps.^17^ Immigration detention is mostly used as a tool to manage migration by speeding up the asylum process. Immigration detention assures compliance with the migration process decision and facilitates efficient deportation after irregular entry, irregular residence or after the commitment of a criminal offence.^14,18^

Even though immigration detention centres mostly do not have a punitive purpose as prisons do, detainees perceive them as punishing and even worse than prisons.^19^ Reliable statistics on how many individuals are currently in detention on a worldwide scale are not available.^20^ In the USA, in 2019, 143 099 forced migrants were arrested, and the average population in detention centres per day amounted to 50 165. On average, detainees spent 34.3 days in so-called US Immigration and Customs Enforcement detention facilities.^21^ In Canada, between 2019 and 2020, 8825 people were detained in total and spend on average 13.9 days in so-called immigration holding centres.^22^ In Australia, 1440 people were detained in immigration detention centres at 31 August 2021; the majority of them (95%) have been detained for more than 31 days at that point, more than a quarter of them (35%) for more than 2 years.^23^ Australia is particularly being criticised for implementing offshore detention facilities, that are inhumane and unsafe for their detainees.^24,25^ In the UK, between April 2019 and March 2020, 23 075 people were detained.^26^ Many other countries implement immigration detention but do not share statistics.^15^

## The association between immigration detention and mental health

Research on the underlying mechanisms through which immigration detention could have an impact on mental health remains scarce. Some detainees fear their safety because of inhumane conditions, criminalisation, and physical and verbal abuse by the officers. They may often experience uncertainty concerning their future and remain isolated the majority of their time, and experience a loss of control.^27–30^ Studies suggest that the deterioration of mental health in detainees may be related to the loss of agency that is reported by detainees. Refugees and migrants who have experienced lengthy asylum processes often feel trapped, as if they were ‘boxed in’, and report being helpless and hopeless.^31^ The confinement of refugees and migrants has been associated with adverse mental health effects.^28–31^ Upon release from detention, a substantial proportion of the detainees report symptoms of depression, anxiety and PTSD. They often withdraw themselves from others, fearing rejection or exclusion.^29,32–34^ The only efficient way to improve the detainees’ mental health is to release them from detention.^35^

Detained children are at an even higher risk of experiencing symptoms of depression, anxiety or self-harm and this may be as a result of the lack of parental support. Their parents are often distressed and emotionally unavailable, and children have no sense of adult protection.^36^ The detention itself, the exposure to potentially traumatic events in detention as well, and the absence of parental support may negatively affect the children's mental health. Because of its adverse effects, the implementation of immigration detention centres has been repeatedly criticised.^15,27,28^ Even though the UNHCR, among others, has urged countries to apply alternative solutions,^15,19^ immigration detention continues to be a widely used method.^15,20,37^

## The current meta-analysis

Von Werthern and colleagues^37^ performed a systematic review of the impact of immigration detention on mental health. They concluded that detained refugees and migrants experience more severe symptoms of anxiety, depression, PTSD and a lower quality of life than non-detained refugees and migrants. Furthermore, symptoms are more severe when the detainees are isolated. Filges and colleagues^18^ applied meta-analytical methods and found preliminary evidence that immigration detention has an independent role in deteriorating mental health. The authors also conclude that the more time refugees and migrants spend in immigration detention, the more accentuated the symptoms become. However, this evidence derives from only two studies that head-to-head compared detained and non-detained refugees and migrants. Owing to ethical considerations, controlled studies on mental health in detained samples are scarce if not non-existant.^33,38^

The current meta-analysis aims to provide an updated systematic review of the existing body of literature and to add to the previously conducted meta-analytical methods by including single-group studies in the analysis. We thereby address the following research question: are refugees and migrants under immigration detention at increased risk for psychiatric disorders, such as anxiety, depression and PTSD, compared with refugees and migrants in community settings or other non-confining environments?

## Method

The execution and reporting of this meta-analysis followed the guidelines as defined in the PRISMA statement.^39^ A drafted protocol for this meta-analysis was pre-registered in the International Prospective Register of Systematic Reviews (PROSPERO), registration number: CRD42020196078.

### Search and selection strategy

A computer-based search was performed using Embase, Medline, Web of Science and Google Scholar.^40^ The formulation of search strings followed the strategy by Filges and colleagues.^41^ The search terms were all related to immigration detention. In Supplementary Table 1 available at https://doi.org/10.1192/bjo.2021.1026, we present the complete search strategy. The reference lists of systematic reviews and meta-analyses that were conducted on the topic before, as well as included studies, were additionally reviewed for eligible studies. Only articles that were written in English, German, French, Spanish or Dutch were considered. The literature search was carried out, independently, by two researchers (I.V. and M.M.), who also independently screened the identified articles’ titles and abstracts to assess their eligibility. If it was ambiguous whether a study was eligible, the study was assessed in full. Based on the inclusion and exclusion criteria, a conclusion was made on the eligibility of the study. Disagreement was resolved through discussion and consensus.

### Inclusion and exclusion criteria

Inclusion was not limited to comparison studies but extended to single-group, prospective and retrospective cohort studies, case–control studies, cross-sectional studies and multiple case series.

Articles were included when the sample consisted of refugee or migrant populations who were in immigration detention either at the time of the study or who were released from immigration detention. For comparison studies, we included studies that contained a refugee or migrant sample that was not in detention either before or during the time of assessment as a control group. Studies were eligible when individuals were in immigration detention in a country other than their home country and when detention had immigration purposes. Studies that reported prevalence rates or mean severity scores on depression, anxiety disorder, PTSD or other psychiatric disorders, assessed through clinical diagnostic interviews or using the validated cut-off score on self-report questionnaires were included.

Articles were excluded when the detention had a punitive purpose solely, and when detention was not depriving the liberty of movement (such as semi-open centres), when they did not report original data or when participants were selected based on the outcome. In cases where data on the prevalence or the mean severity score of psychiatric disorders were missing in articles where it was expected that such data were gathered, the corresponding authors of these particular articles were contacted with the request to share relevant data. Only if the data could not be acquired was the study excluded. We did not implement additional exclusion criteria for the comparison group, as it is challenging to find a suitable control group and approaches in doing so differ among studies.^18^ Studies were not excluded based on methodological quality.

### Assessment of methodological quality

Included studies were assessed on their methodological quality independently by two of the authors (I.V. and M.M.). The methodological quality was assessed using the quality assessment tool recommended by the US Department of Health and Human Services.^42^

### Data extraction and management

Information on the prevalence of depression, anxiety disorders, PTSD or other psychiatric disorders, participant characteristics, detention characteristics, assessment type, time of assessment (during versus post-detention), sample size and research design were extracted, in duplicate, by two researchers (I.V. and M.M.).

### Statistical analysis

The analyses were conducted using the software Jamovi and the *metafor* package for meta-analyses.^43^ We used the random-effects model with 95% CI for data synthesis.^44^ Pooled prevalence rates of anxiety, depression and PTSD were calculated for child/adolescent and adult detained refugees and migrants separately for data derived by self-report and diagnostic interviews.

The *I*^2^ measure was used as a measure for statistical heterogeneity. To explore statistical heterogeneity, moderator analyses were performed with age (child versus adult samples), gender distribution, host country, time of assessment and assessment type as predictors. Publication bias was assessed by means of Kendall's Tau rank correlation test for the assessment of funnel plot asymmetry. Statistical significance was set at *P* < 0.05.

### Theoretical comparison

The pooled prevalence rates of detained refugees and migrants were compared with prevalence rates for non-detained refugees and migrants as reported in the meta-analysis by Henkelmann and colleagues (2020).^9^ Theoretical comparisons were also made with continent-specific prevalence rates of anxiety, depression and PTSD in general adult and child/adolescent populations.

For the purpose of comparability among samples, we choose estimates derived through a diagnostic interview. The estimates on prevalence rates for non-detained refugees and migrants were derived from the three most recent meta-analyses on non-detained refugees and migrants.^9,10,45^ Studies that reported on detained refugees were excluded from the Henkelmann and colleagues (2020) data^9^ and prevalence rates were re-calculated prior to comparisons.

## Results

### Description of studies

**Fig. 1:**
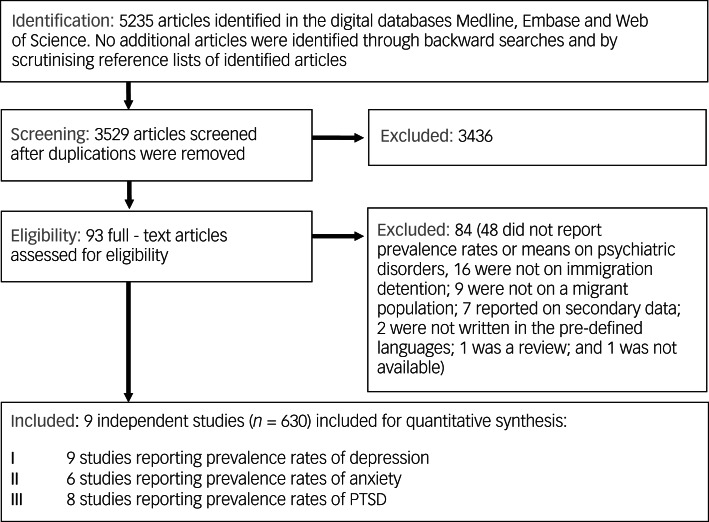
Flow chart on identification, screening and inclusion of eligible articles. PTSD, post-traumatic stress disorder.

The search was performed between July 2020 and 1 October 2020. Overall, we identified 3529 citations after the removal of duplicates. After screening these records, 93 studies were assessed in full text for eligibility. Fig. 1 outlines the search and selection process. Nine independent studies were included in the review, reporting on a total of 630 participants, 522 of them were in immigration detention before or at the time of the study. Key characteristics of the included studies can be found in Table 1.

**Table 1:** Description of studies included in the meta-analysis of the prevalence of psychiatric disorders among forced refugees and migrants in immigration detention

Author (year)	Assessment	*n*, detained	*n*, non-detained	Age, years: mean	Female, %	Country of origin	Host country	Year of assessment	Months in detention^a^	Meta-analysis^b^
Adults										
Coffey et al (2010)^29^	Diagnostic interview post-detention	17	–	42	5.88	Asia, Africa, Europe	Australia	2007–2009	x̄: 38	I, III
Cleveland & Rousseau (2013)^46^	Diagnostic interview/self-report during detention^c^	122	66 (asylum seekers)	31.6	40.96	Africa, Asia, South America and Europe	Canada	2010–2011	x̄: 1	I, II, III
Ehntholt et al (2018)^33^	Diagnostic interview post-detention	35	–	19.1	40	Africa, Asia	UK	2004–2006	x̄: 0.76	I, III
Graf et al (2013)^47^	Diagnostic interview during detention	80	–	29.9	0	Africa, Europe	Switzerland	2007 – 2008	–	I, II, III
Keller et al (2003)^48^	Diagnostic interview during detention	61	–	28	20	Africa, Europe, Asia, South America	USA	2001–2002	x̄: 5	I, II, III
Robjant et al (2009)^49^	Self-report during detention	66	42 (asylum seekers)	29.5	32.88	–	UK	–	x̃: 1	I, II, III
Sen et al (2018)^50^	Diagnostic interview during detention	101	–	31.65	0	Asia, Africa	UK	2014–2015	–	I, II, III
Steel et al (2004)^51^	Diagnostic interview post-detention	14	–	–	64.29	Not disclosed to protect anonymity	Australia	2002–2003	x̄: 28	I, III
Children										
Lorek et al (2009)^52^	Self-report during detention	6	–	–	50	Asia, Jamaica, North America	UK	2006	–	I, II, III
Steel et al (2004)^51^	Diagnostic interview post-detention	20	–	–	40.9	Not disclosed to protect anonymity	Australia	2002–2003	x̄: 28	I, III

a. x̄ = mean, x̄ = median.

b. I. Depression, prevalence; II. Anxiety, prevalence; III. PTSD, prevalence.

c. Assessed using self-report questionnaires; due to literacy problems, clinical interviews were implemented for some of the participants.

The included studies predominantly assessed depression, anxiety and PTSD. For the assessment of depression and anxiety, different instruments were used (see Supplementary Table 2). Supplementary Table 3 provides single-study prevalence rates for other disorders. Country-specific differences in detention policy and approach are provided in Supplementary Table 4.

### Quality assessment

Methodological quality scores for the included studies ranged between 4 and 9.5 (mean 6.3, s.d. = 2.4). The interrater reliability of the quality assessments was high (κ = 0.88, s.e. = 0.04).^53^ In the Supplementary Tables 5 and 6, information on the methodological scores can be found.

### Prevalence rates of depression, anxiety and PTSD in detained refugees and migrants

Pooled prevalence rates were 0.72 for depression (see Supplementary Figure 1), 0.55 for anxiety (see Supplementary Figure 2) and 0.45 for PTSD (see Supplementary Figure 3) for detained refugees and migrants.

For an overview of prevalence rates for depression, anxiety and PTSD in adult and child detainees, see Table 2. Heterogeneity was high in all cases. There was no evidence of publication bias in any of the analyses (see Table 2).

**Table 2:** Prevalence rates of depression, anxiety and post-traumatic stress disorder with 95% CI

	Studies, *n*	Participants, *n*	Prevalence (95% CI)	*I*^2^	Kendall's Tau^a^
Adults					
Depression	9	496	0.68 (0.53–0.83)	93.88***	−0.22
Anxiety	6	429	0.54 (0.36–0.72)	93.85***	0.2
Post-traumatic stress disorder	7	395	0.42 (0.22–0.63)	95.61***	0.52
Children/adolescents					
Depression	2	26	0.83; 0.95^c^	NA***	NA
Anxiety^b^	1	6	1.00	NA	NA
Post-traumatic stress disorder	2	26	0.17; 0.5^c^	NA***	NA

NA, not applicable.

a. Kendall's Tau; rank correlation test for funnel plot asymmetry. A significant correlation is an indication for the presence of publication bias.

b. Only one of the included studies^41^ reported on anxiety prevalence data for children.

c. Only two of the included studies 51,52 reported on depression and PTSD prevalence data for children.

*** *P* < 0.001.

### Prevalence rates of depression, anxiety and PTSD in detained compared with non-detained refugees and migrants

Only two studies met the inclusion criteria for a direct comparison between detained and non-detained refugees and migrants.^46,49^ Therefore, comparative prevalence rates could not be calculated for either depression, anxiety or PTSD.

Among the eligible studies, three included a control group. Keller and colleagues^48^ compared the prevalence rates for anxiety, depression and PTSD in detained versus released non-detained asylum seekers. Their study did not meet our inclusion criteria for comparative analysis, as the control group was in detention at baseline, too. Participants were interviewed twice; at follow-up, the symptoms of the detained sample significantly deteriorated. The detained sample reported significantly higher levels of depression (0.89 *v*. 0.35), anxiety (0.86 *v*. 0.35), and PTSD (0.60 *v*. 0.12) than the non-detained sample.^48^

The two studies that met inclusion criteria reported on refugees and migrants living in community settings as a control group. In both studies, the control group had not been detained before.^46,49^ Cleveland & Rousseau report that significantly more participants in the detained group met the criteria for depression (0.78 *v*. 0.52), anxiety (0.63 *v*. 0.47) and PTSD (0.32 *v*. 0.18) than in the non-detained control group.^46^ Robjant and colleagues compared detained asylum seekers with non-detained asylum seekers living in community settings and former prisoners. The latter control group did not match our inclusion criteria. Detained asylum seekers reported higher mean and prevalence rates for depression (0.76 *v*. 0.26), anxiety (0.72 *v*. 0.50), and PTSD (mean  68.02 *v*. 54.35).^49^

### Moderators for pooled prevalence rates in detained refugees and migrants

Pooled prevalence estimates for depression and PTSD did not differ significantly for adults compared with children (*P* = 0.28 and 0.69, respectively) (for stratified analyses, see Supplementary Table 7). The age of the sample and host country did not have a significant moderating effect on the prevalence rates of depression (*P* = 0.27 and 0.08, respectively), anxiety (*P* = 0.57 and 0.97 respectively) or PTSD (*P* = 0.79 and *P* = 0.23, respectively). The time of assessment (during *v*. post-detention) and the type of assessment (interview *v*. self-report questionnaire) did not have a significant moderating effect on the prevalence rates of depression (*P* = 0.61 and *P* = 0.56, respectively) or PTSD (*P* = 0.53 and *P* = 0.29, respectively). Prevalence rates of anxiety were also not associated with type of assessment (*P* = 0.32) The percentage of female participants in the sample was positively associated with the prevalence rate of depression (*r* = 0.58, *P* = 0.03) but not with the prevalence rate of anxiety and PTSD (*P* = 0.09 and 0.19, respectively).

### Theoretical comparison

Prevalence rates for all three disorders were considerably higher in adults and child/adolescent detained refugees and migrants, notably for depression and anxiety, relative to non-detained refugees and migrants,^8^ (see Supplementary Table 8). Supplementary Table 9 provides an overview of the prevalence estimates for depression, anxiety and PTSD in detained and non-detained refugees and migrants.^9,10,45^

## Discussion

### Summary of main results

The present meta-analysis shows that three out of four detained refugees and migrants experienced depression, more than half of them experienced anxiety, and almost half of them experienced PTSD. Prevalence rates for all three disorders are around twice as high in detained relative to non-detained refugees and migrants. In line with studies on gender differences and depression,^54^ our data shows that estimated prevalence rates were higher in females; however, gender did not have a significant moderating effect on either anxiety or PTSD.

In previously conducted systematic reviews, von Werthern and colleagues^37^ and Filges and colleagues^18^ concluded that immigration detention exacerbates and elicits depression, anxiety and PTSD symptoms. Our current meta-analysis gives further evidence for an independent and negative effect of immigration detention on mental health.

### Immigration detention and the aversive impact on mental health

The literature states that exposure to trauma, especially torture, is linked to PTSD symptoms in a dose-dependent manner, and the severity of pre-migration war-related traumatic events negatively influence trauma-related mental health, such as depression, anxiety and PTSD.^32,55,56^ However, trauma as a stressor cannot solely explain the deterioration of refugees’ and migrants’ mental health. Contextual factors in the hosting country have a significant impact.^57,58^ Symptoms of depression, anxiety and PTSD have been associated with post-migration factors, such as holding a temporary visa, insecurity about visa status, no access to health services and being separated from society.^13,59–61^ Refugees and migrants who are integrated into society or hosted in a supportive environment experience fewer symptoms of depression, distress and PTSD than refugees and migrants separated from society.^27,61,62^ Hence, as expected, prevalence rates of depression and PTSD are higher among non-detained refugees and migrants than among non-refugee or migrant populations.^10,63,64^ Given lack of experimental control, it remains inconclusive whether immigration detention causally elicits or exacerbates anxiety, depression and PTSD symptoms.

Keller and colleagues^48^ published the first study that directly compared symptom scores within-subjects during detention and after being released from detention. They found that depression, anxiety and PTSD symptoms increased with detention length and decreased upon release. Our results did not yield a significant difference between symptoms during detention and post-detention and therefore do not support their finding. In a longitudinal study on asylum seekers holding a temporary protection visa released from immigration detention, Steel and colleagues^34^ found that overall mental health did not improve or even deteriorated further 2 years upon release, compared with after being released from detention. Prospective studies investigating whether mental health improves or deteriorates upon release from detention are scarce. Future research should investigate the development and the content of the PTSD, depression and anxiety symptoms in detained and released refugees and migrants to shed more light on the theoretical explanation of elevated levels of depression, anxiety and PTSD.

### Strengths and limitations

A strength of our meta-analysis is our broad approach (we included studies focusing on detained samples without a control group and studies using different assessment methods). Additionally, we implemented a comprehensive search strategy. We assume, therefore, that all relevant studies on the effect of immigration detention on refugees’ and migrants’ mental health were identified and included in the present meta-analysis.

The methodological score for most studies was good. All included studies were observational; hence, no causal conclusions can be drawn. However, ethical issues rule out the implementation of randomised controlled comparison studies on the impact of immigration detention on mental health. As the included studies used convenience, opportunity or snowball sampling, confounding factors are likely to have an impact on the results of the included studies.

Heterogeneity among studies was high, and the source of the high heterogeneity between studies remains mostly unclear. It is possible that differences between countries, detention centres, visa status or demographic characteristics of the sample accounted for the heterogeneity. Unfortunately, the data reported in the studies was insufficient to specify the impact of those variables, and moderator analyses to investigate their impact were most likely underpowered. Also, it is possible that country-specific policies, such as defining refugees and migrants for an indefinite versus a definite time period, may lead to increased symptoms of depression, anxiety and PTSD. In addition, it is possible that symptoms differ when comparing detention centres managed by private companies compared with federal institutions. In the USA, for example, conditions in private immigration detention centres were less humane compared with federal immigration detention centres.^65^ We had too little data on these potential moderator variables to actually perform such comparisons. Further studies are needed to investigate differences between refugee and migrant samples from different backgrounds and residing in different host countries and institutions.

One source of heterogeneity is the difference in sampling methods between studies. Ehntholt and colleagues^33^ reported mental health data obtained from previously detained refugees who were in a legal process to get compensated for being unlawfully detained as minors. Participants were informed that the mental health assessment aimed to support their legal case. In the studies by Lorek and colleagues^52^ and by Steel and colleagues,^51^ participants responded to an advertisement for free legal assistance to challenge individual detention. Participants in these studies could have exaggerated their symptoms to increase their chances of compensation or being released. It is also possible that the detention's unlawful character increased symptoms of anxiety, depression and PTSD in the study by Ehntholt and colleagues^33^ and that those who reached out for legal assistance in the samples studied by Lorek and colleagues^52^ and Steel and colleagues^51^ are most severely affected by immigration detention. Although the results were not significantly different when those three references were excluded from analysis, further research with greater statistical power is needed to exclude with certainty the possibility that symptoms were not higher in those samples.

Second, our results most likely do not provide a complete picture of the impact of immigration detention on mental health. Very few studies reported on psychiatric disorders other than depression, anxiety or PTSD. Previous research shows that disorders such as personality disorders and psychosis are more prevalent among detained than non-detained refugees and migrants.^47,50^ However, more research on a broader spectrum of psychiatric disorders among refugees and detained migrants is needed to further shed light on this. Also, the pooled prevalence rates are based on samples from only five different countries. However, many other countries implement immigration detention and the conditions vary between detention. Prevalence rates for mental health disorders may be higher in detention centres that are known for their inhumane conditions. Australia, for example, is being criticised for their offshore detention policies.^66^ A study that did not meet inclusion criteria because it was not peer reviewed showed that on Manus Island, 90% of detained asylum seekers report symptoms of depression, anxiety and PTSD.^67^ An investigation by UNHCR revealed that almost 90% of asylum seekers in Papua New Guinea met criteria for depression and anxiety, and almost 80% of them for PTSD.^68^ Other countries are infamous for their inhumane conditions in their detention centres. For example, in immigration detention centres in Libya,^69,70^ detainees endure deprivation of food, water and medical supplies. They often report being sexually assaulted, raped and tortured.^71^ Humanitarian organisations, such as Doctors without Borders or UNHCR, witness adverse effects on the detainees’ mental health. However, to our knowledge, no empirical research on the detainees’ mental health has been conducted yet. It is possible that prevalence rates are higher when studies are conducted in countries known for their inhumane conditions.

Third, among the studies that included a control group,^46,48,49^ the samples differed considerably. Keller and colleagues^48^ administered a mixed design. They studied detained refugees and migrants and compared between- and within-subjects, including a follow-up, at which time part of the sample was released from detention. Their study did not match inclusion criteria. Cleveland & Rousseau^46^ and Robjant and colleagues^49^ included asylum seekers in community settings who had not been held in detention before as a control group. As a result of the scarceness of studies including a control group and the heterogeneity among those who did, pooled prevalence rates for depression, anxiety and PTSD could not be derived to directly compare detained and non-detained samples.

Fourth, using psychiatric concepts such as depression, anxiety and PTSD are limited in investigating the detainees’ symptoms. Those concepts are subject to cultural bias. The instruments used may fail to consider culture-specific expressions of symptoms.^72^ Additionally, some populations tend to report lower symptom scores compared with high-income populations. Those differences are unlikely to decrease the comparability between detained and non-detained refugees and migrants. However, when putting the prevalence rates into broader perspective and comparing them with the prevalence rates of high-income populations or other non-migrant populations, it is important to consider that the symptoms of detainees are most likely to be underreported.^73^ Additionally, in clinical interviews and especially in self-report questionnaires, differentiating between emotional distress as a reaction to adverse circumstances and symptoms of an underlying mental disorder remains challenging.^36^

A final limitation is the lack of comparability between the prevalence data in our sample and the non-detained refugee and migrant sample from the most recent meta-analyses on refugee mental health by Henkelmann and colleagues^9^ and Blackmore and colleagues.^10,45^ These comparisons are not ideal because of the heterogeneity between samples. Such pooled prevalence data can, however, give a first indication for the direction of the impact of immigration detention. Further research is needed to draw more sound conclusions about the mental health of detained compared with non-detained refugees and migrants.

### Implications

In conclusion, immigration detention is associated with independent adverse effects on the mental health of refugees and migrants. Our results strengthen the findings of the previous systematic reviews^18,37,49,74^ that immigration detention harms the mental health of detained refugees and migrants.

Refugees and migrants are a vulnerable sample owing to various pre- and peri-migration factors.^11–13^ Based on our results, it could be argued that immigration detention should no longer be implemented to avoid further mental health deterioration. The aversive effects are likely to outweigh the reasons why some countries employ immigration detention. Countries claim to use immigration detention to guarantee that detainees are present at their proceedings, to ensure that they cannot be a flight risk when they are to be removed, to establish their identity, security status and their health.^15,21,22,75,76^ Receiving countries should use alternative settings to host refugees and migrants. The Council of Europe^77^ and the UNHCR^2^ suggest different arrangements, such as community care, residential facilities or open settings in which forced migrants are required to regularly check-in with authorities. These alternatives are better equipped to host vulnerable populations, such as refugees and migrants, and offer them an adequate home with access to healthcare.^1^ These arrangements serve the reasons for immigration detention mentioned above without leading to further deterioration in the mental health of refugees and migrants or even traumatising them.

## Data Availability

The data that support the findings of this study are available upon request.

## Supplementary material

For supplementary material accompanying this paper visit http://dx.doi.org/10.1192/bjo.2021.1026. click here to view supplementary material
