# Supplementary material for: Prevalence of psychiatric disorders among refugees and migrants in immigration detention: systematic review with meta-analysis
Source: BJPsych Open. 2021 Nov 15;7(6):e204. doi: 10.1192/bjo.2021.1026 (PMC8612016; doi:10.1192/bjo.2021.1026)
Supplement: Supplementary file 1 [file S2056472421010267sup001.docx]

**Supplement: Prevalence of Psychiatric Disorders Among Refugees in Immigration Detention: A Systematic Review with Meta-Analysis**

-- Verhülsdonk, Shahab, Molendijk

**Table S1.** *Search Strategy*

|  | Medline, Embase: Search term |
| --- | --- |
| 1 | (asylum adj1 seek*).mp. [mp=ti, ab, hw, tn, ot, dm, mf, dv, kw, nm, kf, px, rx, an, ui, tc, id, tm] |
| 2 | (Asylumseeker* or Asylum-seeker*).mp. [mp=ti, ab, hw, tn, ot, dm, mf, dv, kw, nm, kf, px, rx, an, ui, tc, id, tm] |
| 3 | Asylum applicant*.mp. [mp=ti, ab, hw, tn, ot, dm, mf, dv, kw, nm, kf, px, rx, an, ui, tc, id, tm] |
| 4 | (Asylum adj1 claim*).mp. [mp=ti, ab, hw, tn, ot, dm, mf, dv, kw, nm, kf, px, rx, an, ui, tc, id, tm] |
| 5 | (Refuge* or Migrant* or Immigrant*).mp. [mp=ti, ab, hw, tn, ot, dm, mf, dv, kw, nm, kf, px, rx, an, ui, tc, id, tm] |
| 6 | Refugees.mp. [mp=ti, ab, hw, tn, ot, dm, mf, dv, kw, nm, kf, px, rx, an, ui, tc, id, tm] |
| 7 | 1 or 2 or 3 or 4 or 5 or 6 |
| 8 | Detention.mp. [mp=ti, ab, hw, tn, ot, dm, mf, dv, kw, nm, kf, px, rx, an, ui, tc, id, tm] |
| 9 | (Depriv* adj2 liberty).mp. [mp=ti, ab, hw, tn, ot, dm, mf, dv, kw, nm, kf, px, rx, an, ui, tc, id, tm] |
| 10 | (Detain or Detained).mp. [mp=ti, ab, hw, tn, ot, dm, mf, dv, kw, nm, kf, px, rx, an, ui, tc, id, tm] |
| 11 | Imprison*.mp. [mp=ti, ab, hw, tn, ot, dm, mf, dv, kw, nm, kf, px, rx, an, ui, tc, id, tm] |
| 12 | Incarcerat*.mp. [mp=ti, ab, hw, tn, ot, dm, mf, dv, kw, nm, kf, px, rx, an, ui, tc, id, tm] |
| 13 | (Reception adj1 cent*).mp. [mp=ti, ab, hw, tn, ot, dm, mf, dv, kw, nm, kf, px, rx, an, ui, tc, id, tm] |
| 14 | (Asylum adj1 cent*).mp. [mp=ti, ab, hw, tn, ot, dm, mf, dv, kw, nm, kf, px, rx, an, ui, tc, id, tm] |
| 15 | (Accomodation adj1 cent*).mp. [mp=ti, ab, hw, tn, ot, dm, mf, dv, kw, nm, kf, px, rx, an, ui, tc, id, tm] |
| 16 | Temporary protection.mp. [mp=ti, ab, hw, tn, ot, dm, mf, dv, kw, nm, kf, px, rx, an, ui, tc, id, tm] |
| 17 | Custod*.mp. [mp=ti, ab, hw, tn, ot, dm, mf, dv, kw, nm, kf, px, rx, an, ui, tc, id, tm] |
| 18 | (Prison* or jail*).mp. [mp=ti, ab, hw, tn, ot, dm, mf, dv, kw, nm, kf, px, rx, an, ui, tc, id, tm] |
| 19 | 8 or 9 or 10 or 11 or 12 or 13 or 14 or 15 or 16 or 17 or 18 |
| 20 | 7 and 19 |
| 21 | remove duplicates from 20 |
|  | Web of Science: Search term | |
| 1 | TS = (Asylumseeker* or Asylum-seeker* or Asylum applicant* or Refuge* or Migrant* or Immigrant* or Refugees) | |
| 2 | TI = (Asylumseeker* or Asylum-seeker* or Asylum applicant* or Refuge* or Migrant* or Immigrant* or Refugees) | |
| 3 | SO = (Asylumseeker* or Asylum-seeker* or Asylum applicant* or Refuge* or Migrant* or Immigrant* or Refugees) | |
| 4 | AB = (Asylumseeker* or Asylum-seeker* or Asylum applicant* or Refuge* or Migrant* or Immigrant* or Refugees) | |
| 5 | AK = (Asylumseeker* or Asylum-seeker* or Asylum applicant* or Refuge* or Migrant* or Immigrant* or Refugees) | |
| 6 | KP = (Asylumseeker* or Asylum-seeker* or Asylum applicant* or Refuge* or Migrant* or Immigrant* or Refugees) | |
| 7 | #6 OR #5 OR #4 OR #3 OR #2 OR #1 | |
| 8 | TS = (Detention or Detain or Detained or Imprison* or Incarcerat* or Temporary protection or Custod* or Prison* or jail*) | |
| 9 | TI = (Detention or Detain or Detained or Imprison* or Incarcerat* or Temporary protection or Custod* or Prison* or jail*) | |
| 10 | SO = (Detention or Detain or Detained or Imprison* or Incarcerat* or Temporary protection or Custod* or Prison* or jail*) | |
| 11 | AB = (Detention or Detain or Detained or Imprison* or Incarcerat* or Temporary protection or Custod* or Prison* or jail*) | |
| 12 | AK = (Detention or Detain or Detained or Imprison* or Incarcerat* or Temporary protection or Custod* or Prison* or jail*) | |
| 13 | KP = (Detention or Detain or Detained or Imprison* or Incarcerat* or Temporary protection or Custod* or Prison* or jail*) | |
| 14 | #13 OR #12 OR #11 OR #10 OR #9 OR #8 | |
| 15 | #14 AND #7 | |

﻿

**Table S2.** *Overview of the instruments used to assess depression, anxiety, and PTSD*

| Authors | Instrument used | |
| --- | --- | --- |
| Cleveland & Rousseau (2013) | HSCL-25ab, HTQc | |
| Coffey et al. (2010) | HSCL-25a, HTQc | |
| Ehntholt et al. (2018) | SCID-IVac | |
| Graf et al. (2013) | CIDIac | |
| Keller et al. (2003) | HSCL-25ab, HTQc | |
| Lorek et al. (2009) | DSRSa, SCASb, R-IES-13c | |
| Robjant et al. (2009) | HADSab, IES-Rc | |
| Sen et al. (2018) | MINI v6.0abc | |
| Steel et al. (2004) | SCID-IVac, K-SADS-PLac | |
| Abbreviations. HSCL-25 = Hopkins Symptom Checklist, HTQ = Harvard Trauma Questionnaire, SCID-IV = Structured Clinical Interview for DSM-IV, CIDI = Composite International Diagnostic Interview, SCAS = Spence Children's Anxiety Scale, R-IES-13 = Revised Impact of Event Scale-13 item HADS = Hospital Anxiety and Depression Scale, MINI v6.0 = Mini International Neuropsychiatric Interview, K-SADS-PL = Kiddie Schedule for Affective Disorders and Schizophrenia, DSRS = Depression Self-Rating Scale  a used to assess depression  b used to assess anxiety disorders  c used to assess PTSD | |

**Table S3.** *Prevalence Rates for Psychiatric Disorders other than Depression, Anxiety, and PTSD among Detained Migrants*

| Authors | Psychiatric disorder | Prevalence |
| --- | --- | --- |
| Graf et al. (2013), N = 80 | Schizophrenia,  Schizophreniform disorder,  Delusional disorder,  Acute and transient psychotic disorders,  Schizoaffective disorders,  Hypomania,  Dysthymia,  OCD,  Dissociative amnesia,  Dissociative anesthesia and sensory loss,  Undifferentiated somatoform disorder,  Hypochondrial disorder,  Persistent somatoform pain disorder | .0625  .0125  .0375  .0125  .025  .0125  .0375  .0125  .025  .0125  .0375  .0125  .075 |
| Sen et al. (2018), N = 101 | Personality disorder,  Autism,  ADHD,  Manic episode,  Mood disorder with psychotic symptoms,  OCD  Hypomania,  Antisocial personality disorder,  Hypomanic symptoms,  Psychotic disorder,  Eating disorder | .3465  .1485  .1386  .099  .099  .0891  .0792  .0792  .0297  .0297  .0099 |
| Steel et al. (2004)  Adults: N = 14  Children: N = 20 | Suicidal ideation,  Self-harm  Suicidal ideation,  Self-harm,  Separation anxiety disorder,  Oppositional defiant disorder | .9286  .3571  .55  .25  .5  .45 |

**Table S4.** Detention policy and approach per country

|  | Australia [2 studies] | Canada [1 study] | United Kingdom [4 studies] | United States of America [1 study] | Switzerland [1 study] |
| --- | --- | --- | --- | --- | --- |
| **Management** | private1 | federal2 | private1 | private1 | federal3 |
| **Grounds for detention** | to prevent unauthorized entry at the border, to establish/verify identity/ nationality (mandatory), for unauthorized entry/stay4 | during asylum process, to establish/verify identity/ nationality, for unauthorized entry/stay, to prevent absconding, to effect removal5 | to effect removal, to establish/verify identity/ nationality, for unauthorized entry/stay, for not respecting non-custodial measures, for unauthorized stay6 | to prevent unauthorized entry at the border, during asylum process, for unauthorized entry/stay, to prevent absconding, for not respecting non-custodial measures7 | to prevent unauthorized entry at the border, to effect removal, for ignoring voluntary removal order, during asylum, to effect removal, for unauthorized stay, to prevent absconding8 |
| **Duration of detention** | indefinite9 | indefinite9 | indefinite9 | indefinite9 | temporary8 |
| **Facilities** | detention centers, offshore processing centers4 | immigration removal centers5 | reception and removal centers, general prisons6 | detention centers, state and national jails, prisons, offshore detention7 | transit zones, prisons, asylum and detention facilities8 |
| **Detention of children** | yes9 | yes | yes9 | yes9 | yes10 |
| **Quality** | offshore facilities are especially criticized due to inhumane conditions4 | criticized for the use of prisons, carceral environments and lack of transparency5 | most heavily criticized in Europe for overcrowding, unsuitable accommodation, lack of access to health care6 | biggest detention system world-wide, criticized for carceral and torture-like environments7 | some centers seem more humane than in other countries; only European country using prisons8 |

**Table S5.** *Quality Assessment of Included Studies*

| **Study** | **1** | **2** | **3** | **4** | **5** | **6** | **7** | **8** | **9** | **10** | **11** | **12** | **13** | **14** | **Total** |
| --- | --- | --- | --- | --- | --- | --- | --- | --- | --- | --- | --- | --- | --- | --- | --- |
| Cleveland & Rousseau (2013) I.V. | **⊕** | **⊕** | **⊕** | **⊕** | **⊕** | **⊕** | **⊕** | **∅** | **⊕** | **∅** | **∅** | **∅** | NAa | **⊕** | 9 |
| Cleveland & Rousseau (2013) M.M. | **⊕** | **⊕** | **⊕** | **⊕** | **⊕** | **⊕** | **⊕** | **⊕** | **⊕** | **∅** | **∅** | **∅** | NAa | **⊕** | 10 |
| Coffey et al. (2010) I.V. | **⊕** | **⊕** | **⊕** | **⊕** | **∅** | **⊕** | **⊕** | **∅** | **⊕** | **∅** | **⊕** | **∅** | NAa | **⊗** | 7 |
| Coffey et al. (2010) M.M. | **⊕** | **⊕** | **⊕** | **∅** | **∅** | **⊕** | **⊕** | **∅** | **⊕** | **∅** | **⊕** | **∅** | NAa | **⊗** | 6 |
| Ehntholt et al. (2018) I.V. | **⊕** | **⊕** | NAb | **⊗** | **∅** | **⊕** | **⊕** | **∅** | **⊕** | **∅** | **⊕** | **∅** | NAa | **⊗** | 4 |
| Ehntholt et al. (2018) M.M. | **⊕** | **⊕** | NAb | **⊗** | **∅** | **⊕** | **⊕** | **∅** | **⊕** | **∅** | **⊕** | **∅** | NAa | **⊗** | 4 |
| Graf et al. (2013) I.V. | **⊕** | **⊕** | NRc | **⊕** | **⊕** | **⊕** | **⊕** | **∅** | **⊕** | **∅** | **⊕** | **∅** | **⊗** | **⊗** | 6 |
| Graf et al. (2013) M.M. | **⊕** | **⊕** | **∅** | **⊕** | **⊕** | **⊕** | **⊕** | **∅** | **⊕** | **∅** | **⊕** | **∅** | **⊗** | **⊗** | 6 |
| Keller et al. (2003) I.V. | **⊕** | **⊕** | **⊕** | **⊕** | **∅** | **⊕** | **⊕** | **⊕** | **⊕** | **⊕** | **⊕** | **∅** | **⊕** | **⊗** | 10 |
| Keller et al. (2003) M.M. | **⊕** | **⊕** | **⊕** | **∅** | **∅** | **⊕** | **⊕** | **∅** | **⊕** | **∅** | **⊕** | **∅** | **⊕** | **⊗** | 7 |
| Lorek et al. (2009) I.V. | **⊕** | **⊕** | NAd | **⊗** | **∅** | **⊕** | **⊕** | **∅** | **⊕** | **∅** | **⊕** | **∅** | NAa | **⊗** | 4 |
| Lorek et al. (2009) M.M. | **⊕** | **⊕** | NAd | **⊗** | **∅** | **⊕** | **⊕** | **∅** | **⊕** | **∅** | **⊕** | **∅** | NAa | **⊗** | 4 |
| Robjant et al. (2009) I.V. | **⊕** | **⊕** | **⊕** | **⊕** | **∅** | **⊕** | **⊕** | **⊕** | **⊕** | **⊕** | **⊕** | **∅** | NAa | **⊕** | 11 |
| Robjant et al. (2009) M.M. | **⊕** | **⊕** | **⊕** | **⊗** | **∅** | **⊕** | **⊕** | **∅** | **⊕** | **⊕** | **⊕** | **∅** | NAa | **⊕** | 8 |
| Sen et al. (2018) I.V. | **⊕** | **⊕** | **⊗** | **∅** | **∅** | **⊕** | **⊕** | **∅** | **⊕** | **∅** | **⊕** | **∅** | NAa | **⊗** | 4 |
| Sen et al. (2018) M.M. | **⊕** | **⊕** | **⊗** | **∅** | **∅** | **⊕** | **⊕** | **∅** | **⊕** | **∅** | **⊕** | **∅** | NAa | **⊗** | 4 |
| Steel et al. (2004) I.V. | **⊕** | **⊕** | NAb | **⊗** | **∅** | **⊕** | **⊕** | **∅** | **⊕** | **∅** | **⊕** | **∅** | NAa | **⊗** | 4 |
| Steel et al. (2004) M.M. | **⊕** | **⊕** | NAb | **⊗** | **⊕** | **⊕** | **⊕** | **∅** | **⊕** | **∅** | **⊕** | **∅** | NAa | **⊗** | 5 |
| **⊕** = yes; **∅** = neutral / don’t know; **⊗** = no  Abbreviations. CD, cannot determine; NA, not applicable; NR, not reported  a no follow up measurement was administered  b the sample was not recruited by the authors, but the authors were contacted by a legal team representing the sample  c response rates were not available to the authors  d participants responded to an advertisement by a charity organisation offering free legal assistance to challenge their detention | | | | | | | | | | | | | | | | |

**Table S6.** *Quality assessment of included studies: mismatch between raters and average total*

| **Study** | **1** | **2** | **3** | **4** | **5** | **6** | **7** | **8** | **9** | **10** | **11** | **12** | **13** | **14** | **Total** |
| --- | --- | --- | --- | --- | --- | --- | --- | --- | --- | --- | --- | --- | --- | --- | --- |
| Cleveland & Rousseau (2013) | **⊕** | **⊕** | **⊕** | **⊕** | **⊕** | **⊕** | **⊕** | **⊗** | **⊕** | **⊕** | **⊕** | **⊕** | **⊕** | **⊕** | 9.5 |
| Coffey et al. (2010) | **⊕** | **⊕** | **⊕** | **⊗** | **⊕** | **⊕** | **⊕** | **⊕** | **⊕** | **⊕** | **⊕** | **⊕** | **⊕** | **⊕** | 6.5 |
| Ehntholt et al. (2018) | **⊕** | **⊕** | **⊕** | **⊕** | **⊕** | **⊕** | **⊕** | **⊕** | **⊕** | **⊕** | **⊕** | **⊕** | **⊕** | **⊕** | 4 |
| Graf et al. (2013) | **⊕** | **⊕** | **⊕** | **⊕** | **⊕** | **⊕** | **⊕** | **⊕** | **⊕** | **⊕** | **⊕** | **⊕** | **⊕** | **⊕** | 6 |
| Keller et al. (2003) | **⊕** | **⊕** | **⊕** | **⊗** | **⊕** | **⊕** | **⊕** | **⊗** | **⊕** | **⊗** | **⊕** | **⊕** | **⊕** | **⊕** | 8.5 |
| Lorek et al. (2009) | **⊕** | **⊕** | **⊕** | **⊕** | **⊕** | **⊕** | **⊕** | **⊕** | **⊕** | **⊕** | **⊕** | **⊕** | **⊕** | **⊕** | 4 |
| Robjant et al. (2009) | **⊕** | **⊕** | **⊕** | **⊗** | **⊕** | **⊕** | **⊕** | **⊗** | **⊕** | **⊕** | **⊕** | **⊕** | **⊕** | **⊕** | 9.5 |
| Sen et al. (2018) | **⊕** | **⊕** | **⊕** | **⊕** | **⊕** | **⊕** | **⊕** | **⊕** | **⊕** | **⊕** | **⊕** | **⊕** | **⊕** | **⊕** | 4 |
| Steel et al. (2004) | **⊕** | **⊕** | **⊕** | **⊗** | **⊕** | **⊕** | **⊕** | **⊕** | **⊕** | **⊕** | **⊕** | **⊕** | **⊕** | **⊕** | 4.5 |
| **⊕** = agreement among raters; **⊗** = disagreement among raters  Abbreviations. NA, not applicable; NR, not reported | | | | | | | | | | | | | | | | |

Methodological quality scores of the included studies ranged between 4 and 9.5 (average = 6.3 [*SD* = 2.4], see **Table S5 and S6**). The inter-rater reliability of the methodological quality assessments was fair (Kappa [] = 0.31).On average, methodological quality score of the included studies was modest to good. Most studies were clear in the formulation of study goals, population, and participation rate. However, hardly any study assessed potential confounding variables or performed follow-up assessments. Obviously not a single study was blinded to participant status.

Two studies did not recruit their sample but were contacted by a legal team representing the subjects to investigate their mental health. One study included participants who responded to an advertisement about free legal assistance. To investigate potential bias from including those studies, the analysis to pool the prevalence rates of depression, anxiety, and PTSD were repeated without those studies (see Table S7). The exclusion of these studies resulted in a non-significant trend indicating that prevalence rates were somewhat lower for depression, anxiety, and PTSD.


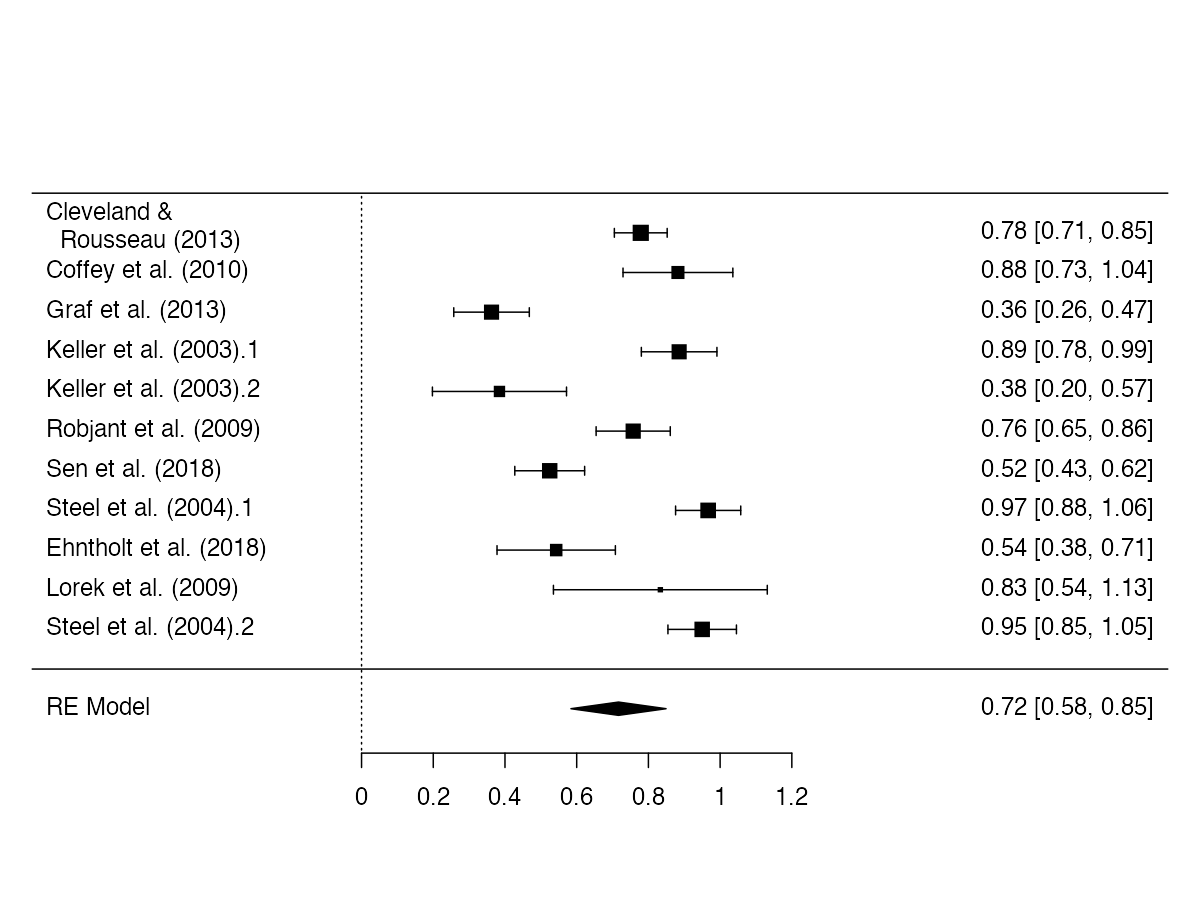


***Figure S1.***Forest Plot of the Prevalence Rates of Depression for Detained Migrants


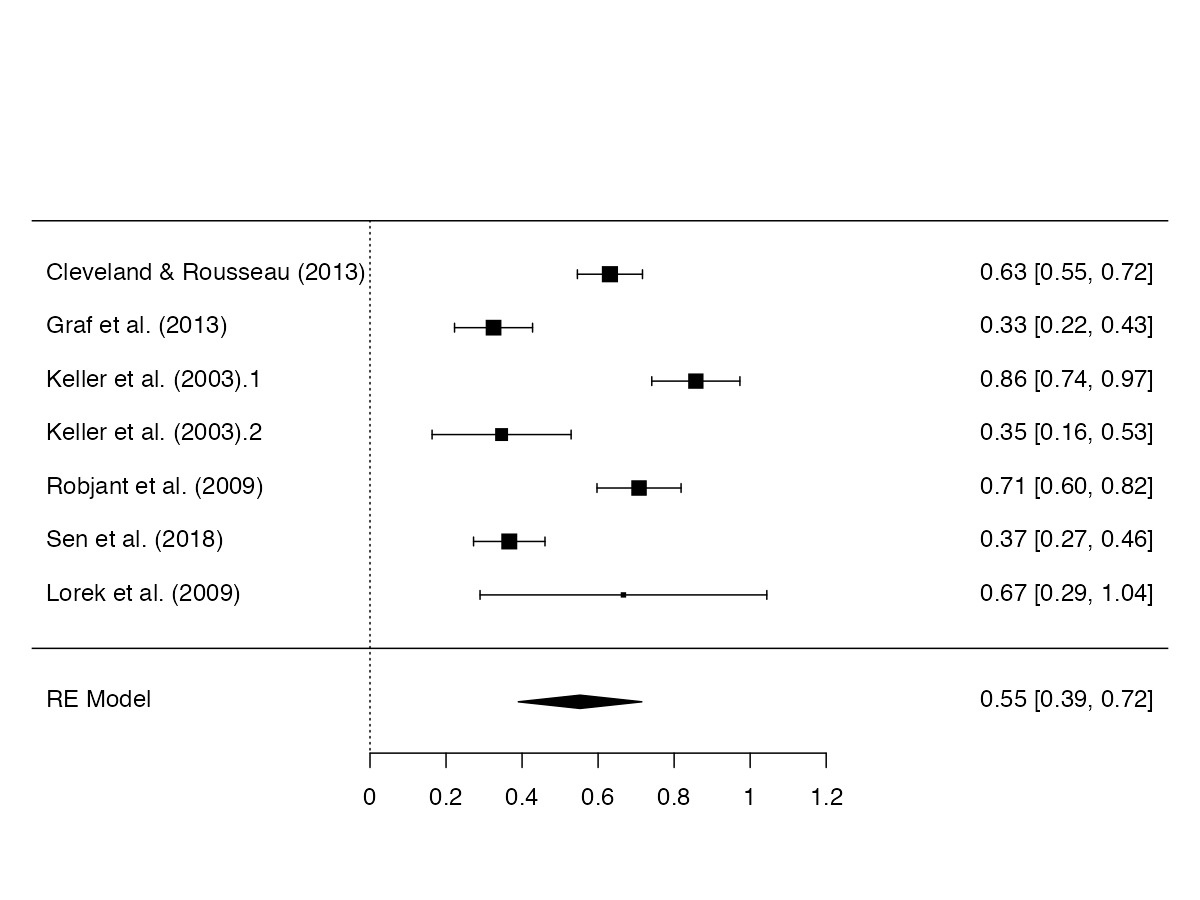


***Figure S2.***Forest Plot of the Prevalence Rates of Anxiety for Detained Migrants


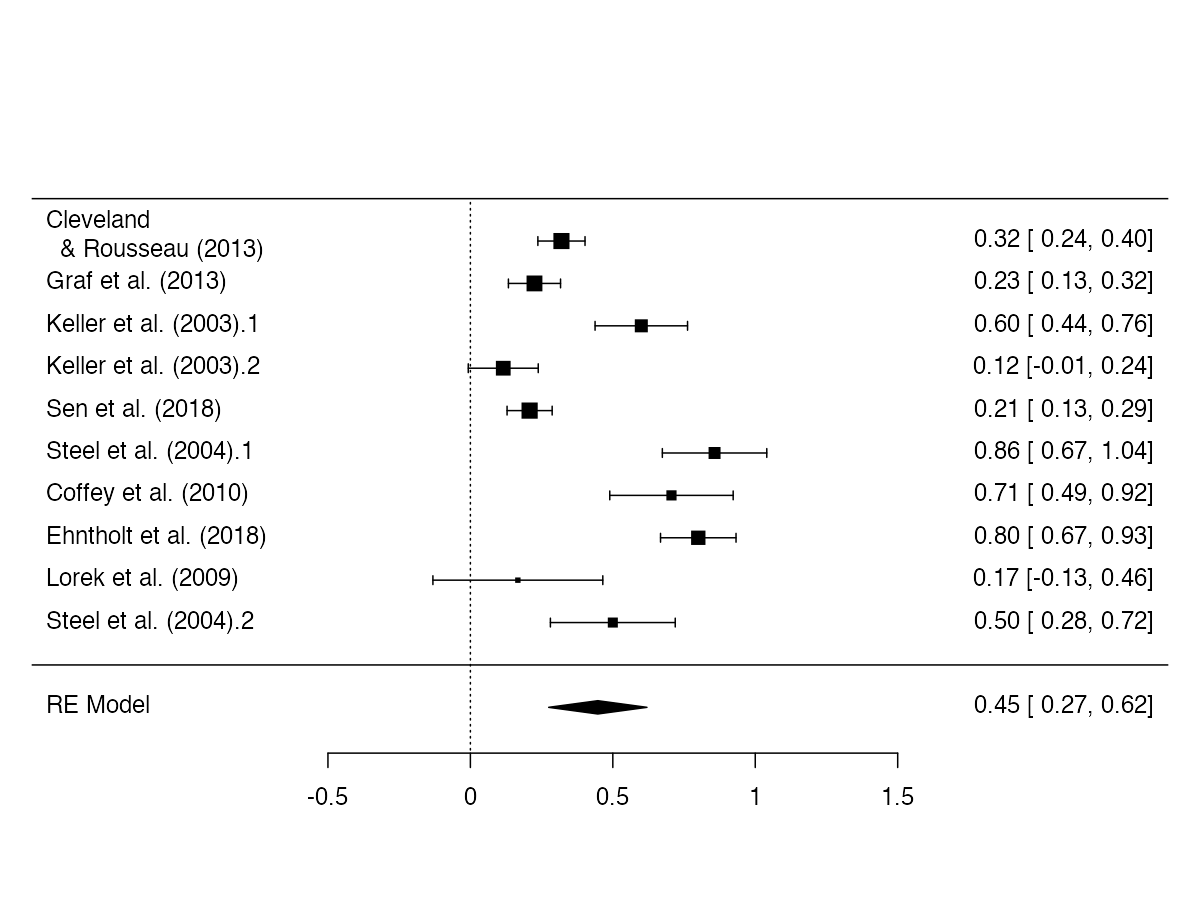


***Figure S3.***Forest Plot of the Prevalence Rates of PTSD for Detained Migrants

**Table S7.** *Pooled Prevalence of Depression, Anxiety and PTSD for Detained Migrants by Time of Assessment (during versus post detention), Assessment Method and without Studies using Convenience Sampling*

|  | *k* | *N* | 95% CI | I2 | Kendall’s Tau a | |
| --- | --- | --- | --- | --- | --- | --- |
| Depression | 11 | 522 | 0.717 (0.584, 0.849) | 93.32*** | -0.273 | |
| During detention | 6 | 410 | 0.682 (0.516, 0.848) | 92.99*** | -0.067 | |
| Post Detention | 5 | 112 | 0.607 (0.525, 0.982) | 94.74*** | -1 | |
| Self-Report Questionnaire | 3 | 194 | 0.774 (0.715, 0.833) | 0 | -0.333 | |
| Diagnostic Interview | 8 | 328 | 0.691 (0.512, 0.871) | 94.83*** | -0.429 | |
| Excluding studies using convenience sampling b | 7 | 447 | 0.657 (0.492, 0.821) | 93.83*** | -0.048 | |
| Anxiety | 7 | 435 | 0.553 (0.39, 0.715) | 92.11*** | 0.143 | |
| During detention | 6 | 409 | 0.585 (0.409, 0.761) | 92.9*** | 0.2 | |
| Post Detention c |  |  |  |  |  | |
| Self-Report Questionnaire d |  |  |  |  |  | |
| Diagnostic Interview | 4 | 242 | 0.475 (0.222, 0.728) | 94.67*** | 0.333 | |
| Excluding studies using convenience sampling b | 6 | 429 | 0.541 (0.363, 0.720) | 93.85*** | 0.2 | |
| PTSD | 10 | 456 | 0.447 (0.275, 0.620) | 94.4*** | 0.244 | |
| During detention | 7 | 378 | 0.409 (0.224, 0.594) | 93.4*** | 0.333 | |
| Post Detention | 3 | 78 | 0.537 (0.111, 0.963) | 95.82*** | 0.333 | |
| Self-Report Questionnaire e |  |  |  |  |  | |
| Diagnostic Interview | 8 | 328 | 0.494 (0.292, 0.696) | 94.71*** | 0.357 | |
| Excluding studies using convenience sampling b | 6 | 381 | 0.349 (0.171, 0.528) | 93.72*** | 0.333 | |
| * p < .05, ** p < .01, *** p < .001  a Kendall’s Tau; rank correlation test for funnel plot asymmetry. A significant correlation is an indication for the presence of publication bias  b Two studies included participants after being contacted by their legal team, one study included participants who responded to an advertisement about free legal aid  c Only one of the included studies (Keller et al., 2003) reported on anxiety prevalence data assessed post detention  d Only two of the included studies (Cleveland & Rousseau, 2013; Robjant et al., 2009) made use of self-reported questionnaires  e Only two of the included studies (Cleveland & Rousseau, 2013; Lorek et al., 2009) made use of self-reported questionnaires | | | | | |

**Table S8.** *Overview of the Prevalence of Psychiatric Disorders among Detained Migrants compared to Non-Detained Refugee and Asylum Seeker Samples*

|  | Prevalence (95% Confidence Interval) | | | | | |
| --- | --- | --- | --- | --- | --- | --- |
| Study | Depression | | Anxiety | | PTSD |  |
|  | Int. | Q. | Int. | Q. | Int. | Q. |
| Current meta-analysis | **0.69** (0.51 to 0.87) | **0.77** (0.72 to 0.83) | **0.48** (0.22 to 0.73) | *No data* | **0.49** (0.29 to 0.70) | *No data* |
| Henkelmann et al. (2020) * | **0.30** (0.22 to 0.40) | **0.39** (0.30 to 0.48) | **0.11** (0.08 to 0.15) | **0.40** (0.26 to 0.56) | **0.27** (0.21 to 0.33) | **0.32** (0.24 to 0.40) |
| Abbreviations.Int. = clinical, diagnostic interview; Q. = self-report questionnaire  * A total of 6 effect-size estimates, derived from 3 original studies, were excluded from the original Henkelmann et al. (2020) data-sets since these were based on populations of adult detained migrants of a mix of adult detained and non-detained migrants. | | | | | | |

**Table S9.** Prevalence rates and 95% CI’s of anxiety, depression and PTSD for adult and child/adolescent detained refugees and non-detained refugees and migrants.

|  | *Adults* | | | *Children/Adolescents* | | |
| --- | --- | --- | --- | --- | --- | --- |
|  | Depression | Anxiety | PTSD | Depression | Anxiety | PTSD |
| Detained refugees and migrants  Current study | **0.7**  (0.53 to 0.86) | **0.51**  (0.34 to 0.69) | **0.42**  (0.22 to 0.63) | **0.78**  (0.52 to 1.04) | *not available* | **0.51**  (0.15 to 0.86) |
| Non-detained refugees and migrants |  |  |  |  |  |  |
| Henkelmann et al. (2020) * | **0.35**  (0.38 to 0.42) | **0.26**  (0.19 to 0.33) | **0.27**  (0.22 to 0.37) | **0.28**  (0.19 to 0.37) | **0.32**  (0.28 to 0.37) | **0.52**  (0.35 to 0.68) |
| Blackmore et al. (2019, 2020) | **0.32**  (0.23 to 0.4) | **0.11**  (0.68 to 0.15) | **0.31**  (0.24 to 0.39) | **0.14**  (0.06 to 0.22) | **0.16**  (0.08 to 0.24) | **0.23**  (0.13 to 0.33) |

* A total of 6 effect-size estimates, derived from 3 original studies, were excluded from the original Henkelmann et al. (2020) data-sets since these were based on populations of adult detained migrants of a mix of adult detained and non-detained migrants.

References

1. Conlon D., Hiemstra, N. Intimate Economies of Immigration Detention: Critical Perspectives. 1st ed. London: Routledge; 2017.
2. Government of Canada. Detention of Foreign Nationals facing Removals [Internet]. [place unknown]: Public Safety Canada; 2021 [updated 2021 May 04; cited 2021 June 07]. Available from: <https://www.publicsafety.gc.ca/cnt/trnsprnc/brfng-mtrls/prlmntry-bndrs/20210708/005/index-en.aspx>
3. Flynn, M., Cannon, C. Immigration detention in Switzerland: A global detention project special report. Geneva: Global Detention Project; 2011; 1-44. Available from: <https://www.refworld.org/docid/545b356c4.html>
4. Global Detention Project (GDP). Country Report: Australia [Internet]. Geneva: GDP. Available from: <https://www.globaldetentionproject.org/countries/asia-pacific/australia>
5. Immigration detention in Canada: progressive reforms and missed opportunities [Internet]. Geneva: Global Detention Project; 2021. Available from: <https://www.globaldetentionproject.org/wp-content/uploads/2021/04/GDP-Immigration-Detention-in-Canada-2021.pdf>
6. United Kingdom immigration detention profile [Internet]. Geneva: Global Detention Project; 2016. Available from: <https://www.globaldetentionproject.org/wp-content/uploads/2016/10/UK-Immigration-Detention-Report.pdf>
7. United States immigration detention profile [Internet]. Geneva: Global Detention Project; 2016. Available from: <https://www.globaldetentionproject.org/wp-content/uploads/2016/06/us_2016.pdf>
8. Switzerland immigration detention data profile [Internet]. Geneva: Global Detention Project; 2020. Available from: <https://www.globaldetentionproject.org/wp-content/uploads/2020/11/Switzerland-Detention-Data-Profile-2020.pdf>
9. United Nations High Commissioner for Refugees (UNHCR). *Global Strategy Beyond Detention.* UNHCR, 2020 (<https://www.unhcr.org/protection/detention/5fa26ed64/unhcr-global-strategy-beyond-detention-final-progress-report-2014-2019.html>)
10. <https://www.streetchildren.org/legal-atlas/map/switzerland/police-roundups/can-children-be-detained-after-being-rounded-up-by-the-police/>
